# Proton Sensing on the Ocular Surface: Implications in Eye Pain

**DOI:** 10.3389/fphar.2021.773871

**Published:** 2021-11-24

**Authors:** Núria Comes, Xavier Gasull, Gerard Callejo

**Affiliations:** ^1^ Neurophysiology Laboratory, Department of Biomedicine, Medical School, Institute of Neurosciences, Universitat de Barcelona, Barcelona, Spain; ^2^ Institut d’Investigacions Biomèdiques August Pi i Sunyer (IDIBAPS), Barcelona, Spain

**Keywords:** ocular surface, pain, ion channels, protons, ocular disease

## Abstract

Protons reaching the eyeball from exogenous acidic substances or released from damaged cells during inflammation, immune cells, after tissue injury or during chronic ophthalmic conditions, activate or modulate ion channels present in sensory nerve fibers that innervate the ocular anterior surface. Their identification as well as their role during disease is critical for the understanding of sensory ocular pathophysiology. They are likely to mediate some of the discomfort sensations accompanying several ophthalmic formulations and may represent novel targets for the development of new therapeutics for ocular pathologies. Among the ion channels expressed in trigeminal nociceptors innervating the anterior surface of the eye (cornea and conjunctiva) and annex ocular structures (eyelids), members of the TRP and ASIC families play a critical role in ocular acidic pain. Low pH (pH 6) activates TRPV1, a polymodal ion channel also activated by heat, capsaicin and hyperosmolar conditions. ASIC1, ASIC3 and heteromeric ASIC1/ASIC3 channels present in ocular nerve terminals are activated at pH 7.2–6.5, inducing pain by moderate acidifications of the ocular surface. These channels, together with TRPA1, are involved in acute ocular pain, as well as in painful sensations during allergic keratoconjunctivitis or other ophthalmic conditions, as blocking or reducing channel expression ameliorates ocular pain. TRPV1, TRPA1 and other ion channels are also present in corneal and conjunctival cells, promoting inflammation of the ocular surface after injury. In addition to the above-mentioned ion channels, members of the K_2P_ and P2X ion channel families are also expressed in trigeminal neurons, however, their role in ocular pain remains unclear to date. In this report, these and other ion channels and receptors involved in acid sensing during ocular pathologies and pain are reviewed.

## 1 Introduction

Physical and chemical stimuli from the environment are sensed by sensory nerve terminals present in the cornea and conjunctiva. Whereas the cornea lies in front of the iris and pupil, the conjunctiva covers the posterior part of the eyelids (palpebral conjunctiva) towards the conjunctival fornix and continues with the anterior part of the sclera until the corneoscleral limbus (bulbar conjunctiva). Both structures are the first line of defense against potential damaging stimuli of the inner eye structures and are covered by a tear film that moistures and lubricates the anterior ocular surface avoiding damage of the corneal epithelium. Acidic insults can reach the ocular surface when we are in contact with exogenous acidic substances. Besides, different infections, allergic or inflammatory conditions can promote an acidic environment in the cornea or the conjunctiva. Moreover, many ophthalmic drugs used as eyedrops are formulated in acidic solutions to be able to solubilize or stabilize the active compound. All these acidic stimuli activate different mechanisms in the ocular surface, mostly ion channels activated by protons in peripheral sensory nerves, which detect and transduce these stimuli to higher brain areas to evoke painful sensations and to induce protective responses. Acidic conditions can also activate ion channels in corneal epithelial, endothelial or conjunctival cells, thus promoting inflammatory states. The mechanisms involved in proton sensing in these ocular structures are reviewed in this report.

## 2 The Ocular Surface

### 2.1 Ocular Innervation

**FIGURE 1:**
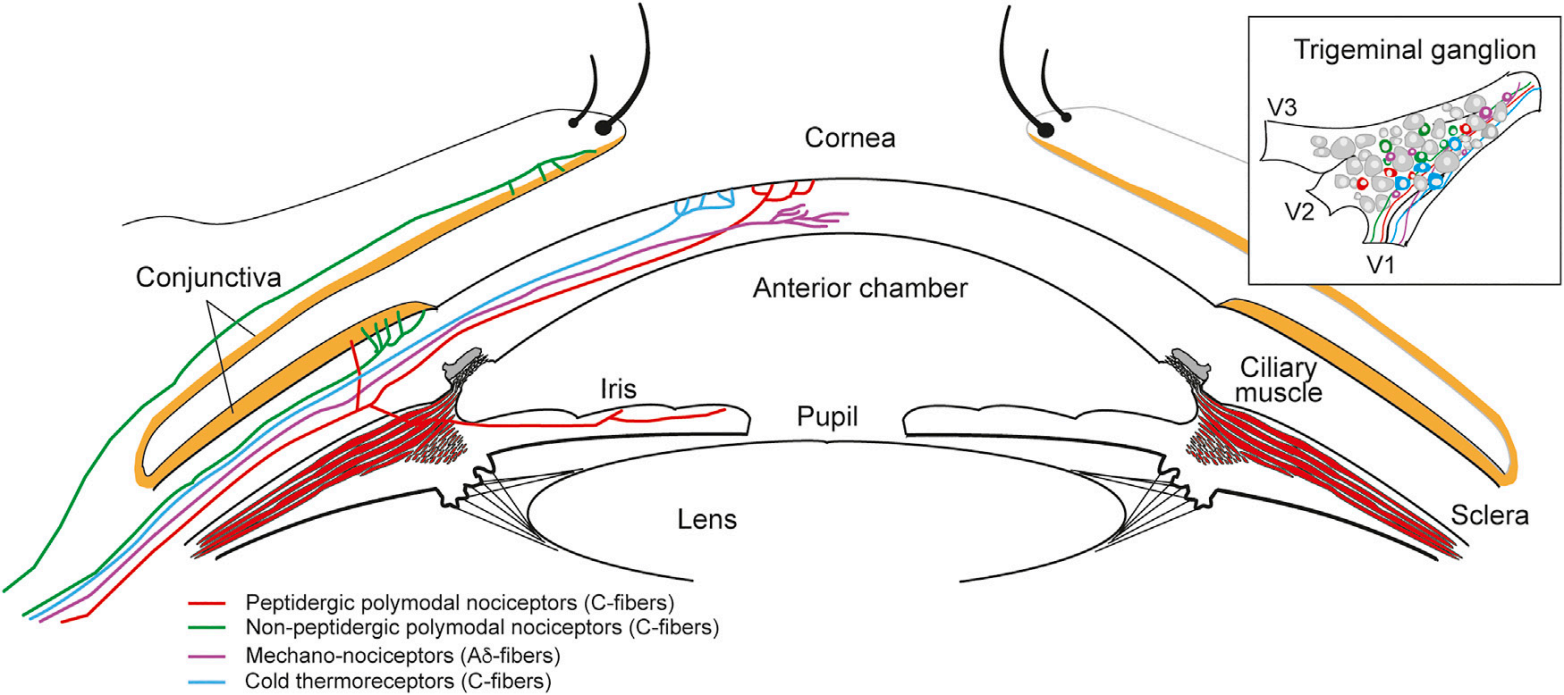
Sensory innervation of the ocular anterior segment. Diagram of the anterior part of the eye showing the different types of nerve fibers innervating the different structures and the ocular surface. Sensory innervation is provided by the ophthalmic nerve arising from the V1 branch of the trigeminal ganglion (upper right inset).

The trigeminal ganglion, through the ophthalmic nerve, provides non-visual sensory innervation to the entire eyeball. Peripheral axons of trigeminal neurons innervate the anterior ocular surface, namely the cornea and conjunctiva, but also the uvea (Figure 1), where they have a critical role in ocular inflammation (Mintenig et al., 1995; Belmonte et al., 1997). Most of the sensory neurons innervating the eye detect mechanical, thermal and chemical stimuli in the noxious range to protect the eyeball, evoking responses to minimize damage and to promote tissue repair. Besides, Edinger-Westphal nucleus localized in the brainstem supplies autonomic parasympathetic innervation of the eye through the oculomotor nerve (ten Tusscher et al., 1994; Reiner et al., 1983). The iris, the ciliary body/ciliary muscle and parts of the iridocorneal angle (uveal trabecular meshwork and scleral spur) are innervated by parasympathetic nerve fibers that synapse in the ciliary ganglion, entering the ocular globe through the short ciliary nerves. In addition, some parasympathetic fibers arrive from the pons through the geniculate ganglion (Petrosal). Later, they synapse in the pterygopalatine ganglion before entering the eye (Ruskell, 1970). Furthermore, sympathetic nerve fibers from the superior cervical ganglion innervate the eyeball through both the long and short ciliary nerves. They innervate the ciliary body (central stroma and stroma of the ciliary processes), the iris and parts of the iridocorneal angle (Figure 1). Contrary, the cornea is innervated almost exclusively by sensory fibers, lacking autonomic innervation.

**FIGURE 2:**
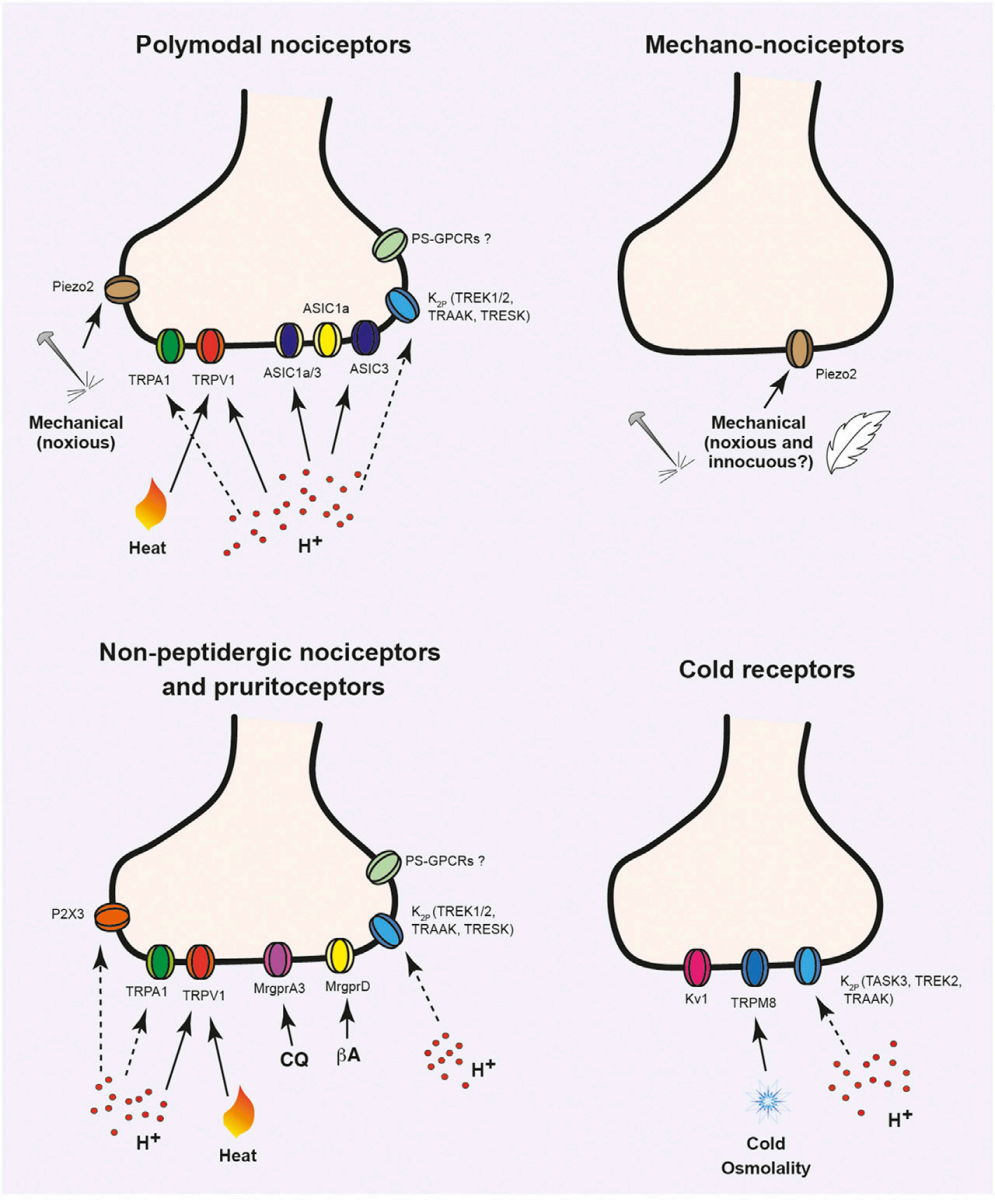
Ion channels activated or modulated by acidic stimuli in ocular sensory neurons. Ion channels expressed in trigeminal sensory neuron terminals innervating the cornea, sclera and conjunctiva. Different types of nociceptive fibers are shown: mechano-nociceptors that respond to high threshold mechanical stimulation; polymodal nociceptors, which can be activated by chemical, mechanical and thermal (noxious heat or cold) stimulation; cold thermoreceptors that express TRPM8 and respond to non-noxious cold stimuli and non-peptidergic sensory neurons, involved in nociception and itch sensitivity. Solid lines indicate direct activation by stimulus. Dashed lines indicate modulation of ion channel activity by protons. CQ, chloroquine; βA, β-alanine.

As mentioned, the ocular surface is densely innervated by trigeminal sensory neurons (Belmonte et al., 2004; Belmonte et al., 2011; Belmonte, 2019), most of them nociceptors (pain sensory neurons; Figure 1). Two main types of nociceptors are present: about 70% are polymodal nociceptors (C-fibers) that respond to mechanical stimulation, extreme temperatures, exogenous chemical irritants and endogenous molecules released by tissue injury. Between 15 and 20% of the nerve fibers are mechano-nociceptors (Aδ-fibers), activated by noxious mechanical forces. Finally, cold thermoreceptors constitute the third population of fibers that innervate the cornea (10–15%), which detect changes in temperature in the non-noxious cold range and regulate basal tearing rate among other functions (Belmonte et al., 2004; Belmonte, 2019). Several ion channels present in the peripheral terminals of these neurons have been characterized and play significant roles in ocular pain (acute, inflammatory or of neuropathic origin), including the sensitivity to protons, as well as in other ocular sensations, such as ocular dryness (Figure 2). A description of some of these ion channels and receptors is detailed in the following sections.

Recent studies using next-generation sequencing techniques have defined 11 subtypes of Dorsal Root Ganglia (DRG) sensory neurons according to different membrane receptors, ion channels, transcription factors and neuropeptides characteristically and similarly expressed (Chiu et al., 2014; Usoskin et al., 2015; Zeisel et al., 2018). The classification of these neurons according to their gene expression patterns and the known roles of these genes on sensory transduction and neuronal excitability, has permitted to define different subgroups of neurons according to their putative function. Therefore, we can differentiate populations of low-threshold mechanoreceptors (touch) and proprioceptors, cold thermoreceptors, heat thermoreceptors as well as peptidergic and non-peptidergic nociceptors activated by high threshold mechanical, thermal or chemical stimuli (Usoskin et al., 2015; Nguyen et al., 2017; Zeisel et al., 2018). Also, specific subpopulations of nociceptors have been described to respond specifically to pruritogens and can be classified as itch receptors or pruritoceptors.

Despite most of the studies have been done in DRG neurons, a few transcriptomic studies in the whole trigeminal ganglia have been done (Flegel et al., 2015; Nguyen et al., 2017; LaPaglia et al., 2018). Trigeminal neurons show a similar distribution of sensory neuron subtypes according to their gene subpopulation markers, which, in general, are similar to those found in the DRG, but specific differences in gene expression are present (Flegel et al., 2015; Nguyen et al., 2017; LaPaglia et al., 2018). A specific transcriptomic study on ocular sensory neurons is still lacking but evidence from different transgenic animal models or from functional studies indicate that some of these neuronal subpopulations of neurons identified in the DRGs or in the TGs neurons specifically innervate different parts of the ocular surface. Specifically, the larger population of sensory neurons innervating the cornea are peptidergic polymodal nociceptors (for review see Belmonte et al. (2011), Belmonte (2019)). At the transcriptomic level, these neurons express distinctive nociceptive markers such as the capsaicin and heat sensitive ion channel TRPV1 (Transient Receptor Potential cation channel subfamily V member 1), the ion channel TRPA1 (Transient Receptor Potential cation channel subfamily A member 1) activated by irritant substances as well as the neuropeptides calcitonin gene-related peptide (CGRP) and substance P (Nguyen et al., 2017). Functional studies have shown that corneal neurons respond to acid, hot temperatures, mechanical stimuli and capsaicin (Chen et al., 1997; López de Armentia et al., 2000; Cabanes et al., 2002; González-González et al., 2017), which is in agreement with their receptor’s expression. Thus, upon stimulation, peripheral terminals of sensory neurons release CGRP and substance P, which are proinflammatory neuropeptides and contribute to neurogenic inflammation and nociceptor sensitization. Although it has not been clearly demonstrated, it is possible that a population of Aδ-fibers constituting “silent” nociceptors innervates the cornea and annex ocular structures. It is thought that these sensory terminals are only activated after local inflammation occurs and the fibers are somehow sensitized. The cellular correlate of these fibers has not been yet established.

Besides polymodal nociceptors, mechanonociceptor neurons (15–20%) only respond to high threshold mechanical stimuli and they express the mechanosensitive channel Piezo2 but do not express neuropeptides (e.g., CGRP) or the cold-sensitive ion channel TRPM8 (Transient Receptor Potential cation channel subfamily M member 8), thus constituting a different subpopulation of corneal neurons besides polymodal nociceptors or cold thermoreceptors (Bron et al., 2014). The last population of sensory neurons innervating the cornea are cold thermoreceptors that express TRPM8. They provide profound innervation of the corneal epithelium and respond to moderate cold stimuli and to changes in osmolarity (Parra et al., 2010; Parra et al., 2014; Quallo et al., 2015). Importantly, these neurons are activated in ocular dryness conditions, when temperature slightly decreases and osmolarity increases due to tear evaporation. Their increase in firing activates a brainstem neuronal loop that regulates basal tearing and blinking rate (Parra et al., 2010; Parra et al., 2014; Quallo et al., 2015). At the molecular and functional levels two populations of TRPM8 have been identified. One presents a high expression pattern of TRPM8 and a lower threshold for activation by cold (González-González et al., 2017; Nguyen et al., 2017), whereas the second one shows a lower expression of TRMP8 and a higher threshold for activation (activation at lower temperatures). This population might co-express other channels like TRPV1 and could be functionally similar to polymodal nociceptors (González-González et al., 2017).

Interestingly, the conjunctiva, presents a different pattern of innervation compared with the cornea. Two neuronal populations innervate this structure but not the cornea: non-peptidergic MAS-related G protein-coupled receptor member D positive neurons (MrgprD^+^) and MrgprA3^+^ sensory neurons (Huang et al., 2018). MrgprD^+^ neurons also express lysophosphatidic acid receptors LPAR3 and LPAR5 and are involved in mechanical pain and skin itch mediated by β-alanine. In the eye, MrgprD^+^ fibers mainly innervate the marginal conjunctiva, a region that contacts with the eye surface during blinking (lid wiper) (Huang et al., 2018). Conversely, MrgprA3^+^ fibers are enriched in medial and lateral conjunctival areas (corners of the eye). These fibers are also activated by histamine, serotonin, chloroquine (an MrgprA3 agonist) and NPFF (that activates MrgprC11), constituting a common pathway for ocular itch (Huang et al., 2016; Huang et al., 2018).

### 2.2 Ion Channels in Ocular Sensory Neurons

The different types of sensory neurons innervating the ocular surface express multitude of ion channels that detect and transduce different physical, thermal or chemical stimuli or that participate in the electrical activity of these neurons (Figure 2). As mentioned earlier, different members of the Transient Receptor Potential (TRP) family of ion channels are present ocular sensory neurons. Particularly, cold thermoreceptors express TRPM8, activated by moderate cold stimuli and menthol (Belmonte and Gallar, 2011). Polymodal nociceptors express TRPV1 and TRPA1 (Belmonte et al., 1991; González-González et al., 2017), purinergic P2X receptors as well as members of the Acid-Sensing Ion Channels, since ASIC1 and ASIC3 currents have been detected in corneal sensory neurons (Callejo et al., 2015). The mechanotransducer channel Piezo2 is present in about 30% of corneal afferent neurons (Bron et al., 2014). Although some expression might exist in some polymodal nociceptors, Piezo2 seems to be mostly restricted to medium- to large-sized sensory neurons positive for neurofilament 200 (NF200) and negative for TRPV1 and CGRP, which suggests its expression in pure mechanonociceptors conducting in the range of Aδ-fibers rather than corneal polymodal nociceptors (Bron et al., 2014; Fernández-Trillo et al., 2020). Moreover, sensory-specific ablation of Piezo2 reduces the percentage of corneal mechanosensitive neurons *in vitro* (Fernández-Trillo et al., 2020). Conjunctival MrgprD^+^ and MrgprA3^+^ afferent neurons also contain TRPV1 and TRPA1 channels that participate in itch stimuli transduction (Huang et al., 2016). These neuronal subpopulations also present a characteristic expression of different ion channels involved in the generation and propagation of action potentials (APs) and in the control of neuronal excitability such as voltage-gated sodium (Na_v_1.7, 1.8, and 1.9), calcium (Cacna1/2/3), potassium (Kcns1, Kcnip4) channels and members of the K_2P_ ion channel family.

Several of these channels are directly activated by protons or modulated by them, thus constituting the transducers for acidic stimuli in the ocular surface (Figure 2). A detailed role of each channel is provided below.

## 3 Proton-Sensing in the Ocular Surface

### 3.1 TRPV1

The vanilloid receptor TRPV1 is one of the best characterized members of the subfamily of the thermosensitive channels TRP. It is a non-selective cation channel permeable to Na^+^ and Ca^2+^ and its activation depolarizes nociceptive sensory neurons (Basbaum et al., 2009). In fact, it has been mainly detected in nociceptors of the trigeminal and the dorsal root ganglia (Clapham, 2003) although they have been found in different brain regions (Tóth et al., 2005). As mentioned before, TRPV1 is a molecular transducer of thermal and chemical painful stimuli, playing a significant role in nociception (Caterina et al., 1997). It is activated by noxious heat, with a thermal threshold activation of >42°C, low pH, voltage and capsaicin, the pungent component responsible for the spiciness of chili peppers (Caterina et al., 1997; Tominaga et al., 1998). TRPV1-mediated thermal sensitivity can be modulated by a variety of components of the inflammatory soup (Tominaga et al., 1998; Pethő and Reeh, 2012). Thus, a variety of endogenous bioactive lipids act as positive allosteric regulators of TRPV1 while proinflammatory agents such as cytokines, prostaglandins, bradykinin and neurotrophins act on their specific receptors modulating TRPV1 through intracellular signaling pathways. TRPV1 activity is essential in the cellular mechanisms by which tissue damage and nociceptor persistent activation can cause acute sensitivity to noxious heat stimuli, thermal hyperalgesia and neurogenic inflammation (Caterina et al., 2000; Szolcsányi and Sándor, 2012). TRPV1 is widely used as a molecular marker for a specific subset of polymodal nociceptors, the small-diameter peptidergic C-fibers characterized by being unmyelinated, slow-conducting and, in most cases, expressing substance P and CGRP (Tominaga et al., 1998).

As mentioned in the previous section, in mammals, the cornea is mainly innervated by three types of afferent sensory neurons: polymodal nociceptors, mechano-nociceptors and cold-sensitive neurons. In this regard, the presence of TRPV1 in the cornea was initially identified in heat-sensitive polymodal nociceptors by topical application of hypertonic saline, acetic acid and capsaicin (Belmonte and Giraldez, 1981; Belmonte et al., 1988; Belmonte et al., 1991; Gallar et al., 1993). Specifically, about 50% of corneal polymodal C-fibers are stimulated by capsaicin in cats (Belmonte et al., 1991; Chen et al., 1997). Subsequently, TRPV1 channel has been detected almost exclusively in small-diameter C axons colocalizing with CGRP and SCGII (secretogranin II) (Kobayashi et al., 2005; Schecterson et al., 2020).

After reporting the dense innervation of the cornea by the ophthalmic branch of the trigeminal nerve (Arvidson, 1977), the molecular profile of corneal trigeminal neurons expressing TRPV1 has been determined by neuronal retrograde tracing. An important percentage of corneal afferent nerve fibers express TRPV1 in different tested animals (González-González et al., 2017). In guinea pig, 45% of corneal sensory fibers are positive for TRPV1 (the molecular marker of polymodal nociceptors), 28% are positive for Piezo2 (the putative marker of mechano-nociceptors) whereas 8% of them express TRPM8 (a marker of cold-sensitive neurons). In addition, no co-expression between TRPV1 and Piezo2 has been detected in this class of nerve fibers but 6% of TRPV1-immunoreactive neurons also expressed TRPM8. The same study has reported that more than 90% of TRPV1^+^ corneal afferents are probably polymodal nociceptors (Alamri et al., 2015). In rat trigeminal ganglion, 37% of corneal afferent sensory neurons has been found to express TRPV1 while around one third of the TRPV1^+^ afferents express substance P and three quarters of these co-expressed CGRP. Therefore, TRPV1 could act in conjunction with substance P and/or CGRP to transduce nociception in corneal sensory neurons (Murata and Masuko, 2006). In contrast, a slightly lower proportion (23%) of rat corneal afferents has been reported to express TRPV1 in another study (Nakamura et al., 2007). In addition to the different animal models used in the studies, this variability in the proportion of sensory neurons expressing TRPV1 could be explained because cholera toxin subunit B, used in most of the mentioned studies, preferentially labels neurons with large cell bodies whereas the retrograde tracer Fluorogold used in the study led by Nakamura is known to label small and large neurons. In addition to neuronal retrograde tracing, immunohistochemistry and double label *in situ* hybridization, the presence of TRPV1 in corneal polymodal nociceptors has been functionally demonstrated with the strong tearing and blinking response evoked by ocular application of capsaicin (Gonzalez et al., 1993; González-González et al., 2017).

The functional effect of capsaicin on TRPV1 in sensory nerve fibers and its capacity to elicit a burning sensation in the eye allowed to suggest that the action of capsaicin could be involved in the perception of painful thermal stimuli *in vivo* (Caterina et al., 1997). Besides, protons can positively modulate the activation of capsaicin-sensitive sensory neurons, enhancing the capsaicin effect (Petersen and LaMotte, 1993). Experimentally, the stimulation of corneal polymodal nociceptors by extracellular protons can be achieved by the application of acidic solution (such as acid acetic solutions) (Chen et al., 1995) or by pulses of a gas mixture with CO_2_ applied to the corneal surface (Belmonte et al., 1999). In the latter case, CO_2_ combines with H_2_O in the tear film covering the cornea resulting in carbonic acid formation which effectively decreases the pH despite the buffering capacity of bicarbonate-containing tears. In parallel, corneal pain has been quantified in humans as a response to the same stimuli with CO_2_ demonstrating that activation of nociceptors expressing TRPV1 by acidic pH could be a cause of pain following tissue injury (Chen et al., 1995). Human subjects identify burning pain and irritation sensation experimentally caused by acidic stimulation (CO_2_ pulses) on the corneal surface, that it is known to recruit polymodal sensory afferents in the cat’s cornea (Acosta et al., 2001a).

Interestingly, TRPV1 can be a target to manage pain perception and it could be useful after an injury or at postoperative level (Weyer-Menkhoff and Lötsch, 2018). Resiniferatoxin (RTX), a potent TRPV1 agonist, strongly activates TRPV1 generating cellular toxicity resulting from an excessive influx of calcium (Olah et al., 2001). When RTX is administered peripherally, it produces reversible analgesia due to the inactivation of nociceptors expressing TRPV1 (Neubert et al., 2003). Subsequent studies have shown that RTX directly infused into the trigeminal ganglion eliminates pain perception as well as neurogenic inflammation. In the rat cornea, a single topical application of RTX reduces capsaicin sensitivity producing transient analgesia for up to 5 days with no adverse side effects observed in histological studies (Table 1; Bates et al., 2010). Therefore, RTX could have the potential to manage acute pain caused by ophthalmic surgeries, and corneal conditions such as abrasions or ulcers. For chronic pain, it remains to be determined whether reapplication of RTX generate longer lasting corneal analgesia. Likewise, treatment with the TRPV1 antagonist capsazepine, prior to allergic challenge, abolishes spontaneous activity and sensitivity to heat of polymodal nociceptors and reduces their firing response to a CO_2_-mediated acidic stimulus (Table 1). It also attenuates the increased blinking rate found in a model for allergic keratoconjunctivitis in guinea pig (Acosta et al., 2013). Because the augmented blinking is considered a nocifensive response to the eye discomfort caused by the high chemical activation of polymodal nociceptors, TRPV1 has been associated to ocular irritation during allergic episodes (Acosta et al., 2013). In a similar way, an increased blinking rate to topical capsaicin has been described in a guinea pig model of keratitis performed by eye exposure to UV radiation (photokeratitis; Table 1). Hence, the discomfort sensation reported by humans after UV irradiation may be the result of sensitization of polymodal nociceptors by the local release of inflammatory mediators and TRPV1 activation (Acosta et al., 2014). In addition to allergic keratoconjunctivitis and photokeratitis, polymodal nociceptors may play a role in other pathologies that affect the cornea like herpes simplex virus keratitis (Gallar et al., 2010) and corneal sensitivity associated to diabetes mellitus (Neira-Zalentein et al., 2011).

**TABLE 1:** Proton-sensing ion channels involved in ocular surface pathologies.

Channel	Disease	Treatment	Effects	Behavioral response	Animal model	References
TRPV1	Ocular pain	Resiniferatoxin (RTX, agonist)	Ca^2+^-induced cytotoxicity	Reduces capsaicin-induced eye wiping	Rat	Neubert et al. (2003)
	Allergic keratoconjuntivitis	Capsazepine (antagonist)	Abolishes nerve fiber spontaneous activity; reduces firing response	Attenuates eye blinking and tearing	Guinea pig	Bates et al. (2010)
	Dry eye disease	siRNA Tivanisiran (SYL1001)	Not tested	Improves tear quality and hyperemia; reliefs ocular discomfort and pain, avoid damage to the ocular surface	Rat	Moreno-Montañés et al. (2018), Fakih et al. (2021)
	Dry eye disease	A784168 (antagonist)	Not tested	Reduces increased eye blinking induced by lacrimal gland excision	Guinea pig	Benitez-Del-Castillo et al. (2016)
	Photokeratitis	Capsaicin (agonist)	Not tested	Increased blinking	Guinea pig	Acosta et al. (2013)
TRPA1	Allergic keratoconjuntivitis	HC-030031 (antagonist)	Reduces mechanical threshold; attenuates responsiveness to CO_2_	Attenuates eye blinking	Guinea pig	Bates et al. (2010)
	Corneal injury and inflammation	TRPA1^−/−^ Knockout mice	Decrease macrophage infiltration; stromal neovascularization and fibrosis	Not tested	Mouse	Katagiri et al. (2015)
ASIC3	Allergic keratoconjuntivitis	APETx2 toxin (antagonist)	Not tested	Reduces allergen-induced blinking	Rat	Callejo et al. (2015)
	Dry eye disease	APETx2 toxin (antagonist)	Not tested	No effect on acid-induced blinking	Rat	Callejo et al. (2015)

A significant number of TRPM8-expressing sensory neurons also express TRPV1 and this channel could be involved in cold nociception of the cornea enhancing excitability of TRPM8^+^ cells (Li et al., 2019). TRPV1 could also act as a pharmacological target for the treatment of dry eye disease (DED), characterized by tear instability, ocular dryness, irritation, itch, pain and visual disturbances, and commonly linked with ocular surface inflammation (Messmer, 2015). Co-expression between TRPV1 and TRPM8 channels is increased in corneal cool cells of the experimental rat model for DED performed by lacrimal gland excision (LGE), that shows enhanced sensitivity to capsaicin (Hatta et al., 2019). Besides, different studies propose that TRPV1 could intervene in the increased nocifensive response associated with DED in rats treated with the LGE procedure (Bereiter et al., 2018). In this sense, mRNA expression of TRPV1, TRPA1, ASIC1, and ASIC3 is upregulated in the ophthalmic division of the trigeminal ganglion of a mice model of chronic DED (Fakih et al., 2021). Interestingly, instillation of capsazepine not only inhibits the aforementioned genetic upregulation but also reliefs corneal neurosensory symptoms and reduces anxiety-linked behaviors that characterize severe DED. Similarly, TRPV1 protein levels have been found increased in the trigeminal ganglion of a rat model of DED, in which the channel is involved in the enhanced nocifensive responses (Bereiter et al., 2018). Taken together, TRPV1 antagonists could be potential analgesics for DED treatment (Fakih et al., 2021). Likewise, topical administration of tivanisiran (formerly named SYL1001), a siRNA designed to silence the expression of TRPV1, improves tear quality, hyperemia, ocular pain and discomfort characteristic of DED (Moreno-Montañés et al., 2018). Thus, Phase I and II clinical trials have already determined the most effective doses for its therapeutic use to alleviate DED symptoms (Benitez-Del-Castillo et al., 2016). In the same sense, topical application of A784168, a potent antagonist of TRPV1, diminishes the blink rate in a model of chronic tear deficiency in guinea pig (Masuoka et al., 2020).

### 3.2 TRPA1

TRPA1, the only member of the subfamily of ankyrin TRP channels described, is a voltage-dependent, non-selective channel permeable to Ca^2+^, Na^+^, and K^+^. TRPA1 is expressed in sensory neurons that innervate skin, intestinal and pulmonary epithelium, inner ear hair cells and olfactory epithelium, among others (Basbaum et al., 2009). It has a similar main structure as TRPV1 with some specific features such as the presence of a long-terminal ankyrin domain including the regions that confer the thermal and chemical sensitivity (Cordero-Morales et al., 2011) and a TRP-like domain after the S6 transmembrane segment instead of the standard TRP motif. TRPA1 channel contributes to the perception of a great variety of chemical substances that causes pain manifested as burning, skin and eye irritation as well as thermal and mechanical hypersensitivity (Bandell et al., 2004). Reactive chemicals that activate TRPA1 include allyl isothiocyanate (AITC) (the pungent compound found in horseradish, mustard oil and wasabi), cinnamaldehyde (the organic compound responsible for the characteristic taste and smell of cinnamon), allicin (from garlic extract) and diallyl disulfide (from onion) (Logashina et al., 2019). TRPA1 is also activated in response to noxious cold temperatures (<17°C) and endogenous agents such as reactive oxygen species (Logashina et al., 2019). Moreover, it senses environmental irritants such as tear gas, acrolein from air pollution and tobacco smoke and endogenous proalgesic and proinflammatory agents (Bautista et al., 2006; Lindsay et al., 2014). In addition, TRPA1-deficient mice show a significant reduction in painful responses to formaldehyde and 4-hydroxynonenal, which are aldehydes that activate TRPA1 (Macpherson et al., 2007). Furthermore, TRPA1 has been associated to chronic itch (Wilson et al., 2013) and hypersensitivity in different experimental models of persistent inflammatory pain (Dai et al., 2007; Lennertz et al., 2012). Thus, TRPA1 could have a great potential as analgesic and anti-inflammatory target to treat pathologies such as different types of dermatitis (Liu et al., 2013; Oh et al., 2013) and migraine (Materazzi et al., 2013).

TRPA1 is expressed in approximately 35% of the sensory neurons of the trigeminal ganglion (Jordt et al., 2004). In the mouse cornea, TRPA1 channel is mainly expressed in medium-diameter myelinated Aδ-fibers where it colocalizes with neurofilament protein NF200 and secretogranin 3 (SCG3) although it is also present in C-fiber nociceptive sensory neurons (Figure 2; Kobayashi et al., 2005; Schecterson et al., 2020). Similar to TRPV1, TRPA1-related mechanisms play a key role in the persistent tear reduction and symptoms of ocular discomfort observed in the rat model of DED (Katagiri et al., 2015). In addition to this, pretreatment with a TRPA1 antagonist (HC-030031) before the allergic challenge reduces the mechanical threshold of polymodal nociceptors, tend to attenuate the enhanced response de CO_2_ and reverse the enhanced blinking in an animal model of allergic keratoconjunctivitis (Table 1; Acosta et al., 2013). With HC-030031 treatment, as well as in TRPA1^−/−^ knockout mice, it has been reported a decrease in macrophage infiltration, stromal neovascularization and corneal fibrosis in a mouse model of corneal injury. Inhibition of the TGF-β1 signaling pathway in fibroblasts with the loss or blockade of TRPA1 would explain, in part, its role in corneal repair. Therefore, TRPA1 represents a potential therapeutic target for corneal lesions and ocular infections associated to inflammatory fibrosis that can lead to vision loss if not treated properly (Okada et al., 2015).

### 3.3 Acid-Sensing Ion Channels

The Acid-Sensing Ion Channel (ASIC) family belong to the ENaC/Degenerin (DEG) ion channel superfamily which in rodents is composed by at least six different subunits (ASIC1a, ASIC1b, ASIC2a, ASIC2b, ASIC3, and ASIC4) encoded by four genes (accn1-4) (Kellenberger and Schild, 2015). In humans, this ion channel family is expanded by the expression of three and two splice variants for ASIC3 and ASIC4, respectively. However, expression of ASIC1b mRNA has never been identified. Evidence using x-ray crystallography (Jasti et al., 2007) and atomic force microscopy (Carnally et al., 2008) have revealed that functional ASIC channels are arranged as homo- and heterotrimeric channels where specific subunit composition confer different biophysical and pharmacological properties to an ASIC trimer (Hesselager et al., 2004). They are voltage-independent, ligand-gated cation channels mainly permeable to Na^+^ (although ASIC1a homomers are also permeable to Ca^2+^ (Waldmann et al., 1997) activated by extracellular protons and different nonproton ligands (Kellenberger and Schild, 2015; Vullo and Kellenberger, 2019). Not all ASIC subunits can form proton-gated functional channels, for instance, ASIC2b and ASIC4 do not form proton-sensitive homomeric channels but the can interact with other ASIC subunits modulating their ion channel properties and kinetics in response to extracellular acidosis (Hesselager et al., 2004). All ASIC subunits have been detected in DRG and TG neurons from mouse and human tissues (although ASIC4 shows a weak expression) (Zeisel et al., 2018; Nguyen et al., 2017; Flegel et al., 2015; Hockley et al., 2019; Schuhmacher and Smith, 2016), including neurons that innervate the cornea in mice (Figure 2; Callejo et al., 2015). Due to their ability to detect increasing proton concentrations in the extracellular environment, they have been involved in many physiological and pathological processes such as synaptic plasticity, learning and memory, fear conditioning, pain, migraine, epileptic seizures and ischemic stroke (Wemmie et al., 2013; Deval and Lingueglia, 2015; Dussor, 2015; Kellenberger and Schild, 2015; Lee and Chen, 2018; Vullo and Kellenberger, 2019).

As mentioned above, a decrease in pH in the tear film covering the anterior ocular surface induces a nociceptive response in animal models and irritation and burning sensation, and to a lesser extent stinging pain, in humans (Chen et al., 1997; Belmonte et al., 1999; Acosta et al., 2001b; Feng and Simpson, 2003; Callejo et al., 2015). These sensations are evoked by the activation of polymodal nociceptive fibers innervating the cornea and conjunctiva (Chen et al., 1995; Belmonte et al., 1999). Initial studies performed to characterize the molecular identity of acid sensors in the cornea demonstrated that after CO_2_-mediated acidic stimulation with CO_2_ to the cat cornea, 50% of polymodal fibers responding to acid also responded to the TRPV1 agonist capsaicin. These fibers were also blocked by the TRPV1 antagonist capsazepine (Chen et al., 1997), indicating the role of this ion channel in the detection of proton ion concentrations in the cornea. However, the existence of acid-sensitive polymodal fibers that do not respond to capsaicin, suggests the functional expression of others ion channels/receptors capable of detecting extracellular acidosis in the anterior ocular surface. Besides TRPV1, ASICs have been identified as detectors of acidic stimuli in sensory neurons innervating the cornea (Figure 2; Callejo et al., 2015). TRPV1 and ASIC channels differ in their pH sensitivities, whereas TRPV1 is activated at lower pH values (pH 6.4 or below), ASIC channels can detect moderate changes in pH (between pH 7.4 and 6). Moderate acidic stimulation (pH = 6.6) induces depolarization and action potential (AP) firing in a subpopulation of corneal neurons in culture (Callejo et al., 2015). This pH-evoked neuronal firing is abolished by the pretreatment with specific ASIC antagonists such as toxins PcTx1 and APETx2, which inhibit homomeric ASIC1a channels and ASIC3-containing channels, respectively. It is worth mentioning that only 14% of neurons that respond to acid are blocked by PcTx1, whereas APETx2 abolishes the AP firing of the remaining acid responders (86%). This difference could be explained by the expression of different ASIC subunits in the same corneal neuron and a wider range of inhibition of APETx2, which blocks all ASIC3-containing channels. Accordingly, voltage-clamp recordings of corneal neurons in response to moderate acidic pH showed currents with biophysical and pharmacological characteristics of homomeric ASIC1a, homomeric ASIC3 and/or heteromeric ASIC1/3 channels. Moreover, the application of moderate acidic solutions in the rat cornea induces nocifensive behaviors (blinking and scratching) that can be partially prevented with selective and non-selective ASIC antagonists (Callejo et al., 2015). In contrast, the application of a specific ASIC3 agonist (2-guanidine-4-methylquinazoline, GMQ), that can activate ASIC3 at physiological pH, enhances the AP firing rate of corneal sensory fibers and increases the blinking and tearing rate in guinea pigs (Callejo et al., 2015). Altogether, these data suggest that ASIC channels are important proton sensors in the ocular surface, where they are functionally expressed, and crucially participate in the detection of moderate acidifications applied to the cornea and the consequent transduction to painful sensation.

ASIC channel inhibition has been proven effective to ameliorate pain in different animal models of inflammation, where specific inhibition leads to a reduced nocifensive behavior in animals after mechanical and chemical stimulation of the inflamed tissues (Deval et al., 2008; Karczewski et al., 2010; Walder et al., 2010; Deval et al., 2011). Particularly in the eye, ASICs have been involved in a model of allergic keratoconjunctivitis (Table 1; Callejo et al., 2015). In this model, the inhibition of ASIC3-containing channels by APETx2 did not prevent the nocifensive behaviors triggered by the application of an acidic solution (pH 5) on the ocular surface, however, APETx2 treatment reduced the blinking rate of animals exposed to the allergen when a solution at physiological pH was applied. Moreover, whole-cell recordings of labelled TG corneal neurons derived from these animals showed an increase in ASIC current density that was partially inhibited by APETx2 (Callejo et al., 2015). Taking together, these results indicate that ASIC channels play an important role in the development of ocular inflammation and sensitization after an allergic challenge.

### 3.4 Two-Pore Domain Potassium Channels (K_2P_s)

The family of K_2P_ K^+^ channels was the last one identified and described, which has 15 members grouped into six subfamilies (TWIK, TREK, TASK, TALK, THIK, and TRESK) based on sequence and functional similarities (Enyedi and Czirják, 2010). The first K_2P_ channel identified was TWIK1, for Tandem of pore domains in a Weak Inward-rectifying K^+^ channel. Now, this subfamily also contains TWIK2 and KCNK7 (K_2P_7.1). The TREK (TWIK-RElated K^+^ channel) subfamily contains TREK1, TREK2, and TRAAK (TWIK-Related Arachidonic acid Activated K^+^) channels. Members of this subfamily are activated by arachidonic acid, polyunsaturated fatty acids (PUFAs), volatile anesthetics, and pain-related stimuli. The TASK (TWIK-related Acid-Sensitive K^+^ channel) subfamily contains TASK1, TASK3, and TASK5 (K_2P_15.1, KCNK15), and these channels have the common property of being inhibited by extracellular acidification. The TALK (TWIK-related ALkaline pH-activated channel) subfamily includes TALK1, TALK2 (K_2P_17.1, KCNK17) and TASK2 and they have an important role in sensing extracellular alkaline pH. THIK1 and THIK2 conform the THIK (Tandem pore domain Halothane-Inhibited K^+^) channel subfamily and both channels are inhibited by halothane. The last subfamily identified was TRESK (TWIK-RElated Spinal cord K^+^) that has only one member, TRESK (K_2P_18, KCNK18), with the lowest structural and functional similarity to other K_2P_ channels. This channel is the only one in the family being regulated by the intracellular Ca^2+^ concentration through calcineurin-mediated dephosphorylation.

The main role attributed to K_2P_ channels in most cell types is the regulation of membrane potential, as they constitute the leak of potassium through the plasma membrane. Therefore, they are commonly refereed as leak or background potassium channels and their function, together with the Na^+^/K^+^ pump, helps to set the resting membrane potential. K_2P_ channels are the main sustained K^+^ conductance that establish the resting membrane potential in neurons, influencing neuronal excitability over a wide range of membrane potentials, especially between resting and action potential threshold, and shaping the duration, frequency and amplitude of the action potential. Basic biophysical properties of this family of channels, regulation, and interaction with other proteins are reviewed elsewhere, including some comprehensive and extensive reviews (Enyedi and Czirják, 2010; Busserolles et al., 2019).

Almost all K_2P_ channels are expressed in DRG and TG neurons but the relative expression of each channel varies between different neuronal populations and species. In humans, the most prevalent channels in DRG and TG are THIK-2, TASK1 and TWIK1, followed by TREK1, TASK2 and TRESK (Flegel et al., 2015). Other studies found TRESK as the most expressed channel in human TG (Medhurst et al., 2001; LaPaglia et al., 2018). In mouse and rat sensory neurons, TRESK, TRAAK, TREK2, TREK1, TWIK1, and TWIK2 are the most highly expressed channels although relative expression may vary between studies. Interestingly, mutations in TRESK have been involved in pain derived from familial migraine with aura (Lafrenière et al., 2010). This effect is thought to be mediated by non-functional homomeric TRESK channels and heteromeric TRESK/TREK1 or TRESK/TREK2 channels, which enhances trigeminal nociceptors excitability and triggers migraine pain (Royal et al., 2019).

As mentioned, it is known that sensory neurons in the trigeminal ganglia express some of these channels but, at the ocular level, no detailed characterization of the channel types expressed in sensory neurons exist to date. Nevertheless, transcriptomic data indicates that TRESK is enriched in TG compared to DRGs and the presence of TREK1 and TREK2 has also been shown in the TG (Yamamoto et al., 2009; Nguyen et al., 2017; LaPaglia et al., 2018). In particular, TREK1, TREK2 and TRAAK are expressed in small-medium diameter trigeminal neurons (likely nociceptors) which show a significant overlap with TRPV1 expression (Yamamoto et al., 2009). In contrast, poor colocalization of these channels is shown with TRPV2 or TRPM8 in trigeminal neurons, with only small colocalization of TREK1 and TRPM8 in some cells (Yamamoto et al., 2009). In this sense, transcriptomic studies have shown that TRPM8-positive trigeminal neurons express TASK3 and, to a lesser extent, TREK1 (Morenilla-Palao et al., 2014). Therefore, it is likely that nociceptive and thermoreceptive sensory neurons specifically innervating the ocular surface present K_2P_ expression. The members of the TREK subfamily of channels are modulated by changes in pH. The most studied channel, TREK1, in addition to its activation by arachidonic acid and mechanical stimuli, it is also modulated by changes in pH. Intracellular acidification activates the channel and, in addition, enhances channel activity in response to other stimuli, such as membrane stretch (Maingret et al., 1999). Also, extracellular stimuli that induce a decrease in intracellular pH such as bicarbonate (HCO_3_^−^) or CO_2_, produce the same effects (Chen et al., 1997; Lee and Chen, 2018). Whether CO_2_ application to the ocular surface produces an intracellular acidification in sensory nerve terminals it is not known. Nevertheless, if this occurs, TREK1 activation would hyperpolarize the terminal, thus preventing action potential firing. Nevertheless, extracellular acidification strongly inhibits TREK1 and activates TREK2, another closely related channel (Sandoz et al., 2009). These effects are due to histidines H126 and H151 in the extracellular loop of TREK1 and TREK2, respectively, that act as proton sensors. This extracellular regulation of TREK1 would depolarize the cell by inhibiting its potassium current. This might occur in the ocular surface when acidic sensitivity is tested by application of low pH solutions or acetic acid (Belmonte et al., 1991; Callejo et al., 2015). TRESK is also regulated by pH and both extracellular and intracellular acidification inhibit the channel current while alkalinization slightly enhances the channel activity (Sano et al., 2003; Callejo et al., 2013). Despite no clear demonstration is available to date, it is possible that combined inhibition of TREK1 and TRESK by extracellular acidification of the ocular surface reduces channels activity and promotes nociceptor terminals depolarization and firing or, at least, facilitates their activation by other stimuli.

K_2P_ channels TASK3, TREK1, and TASK2 have been also found in cold-sensitive TRPM8^+^ sensory neurons (Morenilla-Palao et al., 2014). The ocular surface, particularly the cornea, present a high innervation by these neurons which, in addition to detect changes in temperature in the cold range, are involved in the regulation of basal tearing rate and the detection of osmolarity changes and ocular dryness (Parra et al., 2010; Belmonte and Gallar, 2011; Quallo et al., 2015). As mentioned before, two subclasses can be distinguished in the population of corneal cold thermoreceptor neurons: a larger population of low-threshold cold thermoreceptors (high TRMP8 expression) and a smaller population of high-threshold cold thermoreceptors (low TRPM8 expression). This last population comprises about 30% of the ocular cold-thermoreceptors and are silent until strong cooling activates them, probably acting as cold nociceptors (Belmonte, 2019). TASK3 is highly expressed in about 30% of these neurons and its activity is highly sensitive to acidification (Kim et al., 2000). The sensitivity of TRPM8 sensory neurons to cold or to TRPM8 agonists (e.g., menthol) is enhanced in the absence of TASK3 or by inhibiting the channel with an acidic solution (pH 6). Interestingly, deletion of TASK3 in mice eliminates the population of high-threshold cold thermoreceptors, indicating that the channel plays a significant role in setting the temperature threshold of these neurons. Therefore, TASK3, together with Kv1, can be acting as a brake in excitability, dampening the sensitivity to cold temperatures of high-threshold, cold-sensitive nociceptive neurons (Madrid et al., 2009; Morenilla-Palao et al., 2014). Whether a simple pH change, without the temperature drop, can activate these neurons has not been tested.

### 3.5 Other Receptors

#### 3.5.1 Proton-Sensing G Protein-Coupled Receptors

On top of the proton-activated ion channels described above, the sensitivity to acid has been described for other ion channels and receptors. Several G protein-coupled receptors (GPCRs) engage heteromeric G proteins in response to acidic stimuli and were termed accordingly as proton-sensing GPCRs (PS-GPCRs). The group of PS-GPCRs is formed by six receptors; the initially described GPR4, GPR65 (TDAG8, T-cell death-associated gene 8), GPR68 (OGR1, Ovarian cancer G protein-coupled receptor 1), and GPR132 (G2A) (Ludwig et al., 2003; Murakami et al., 2004; Wang et al., 2004), together with the recently identified GPR31 and GPR151 (Mashiko et al., 2019). Conserved histidines localized in their extracellular domain confer them the ability to be activated by acidic stimuli in the pH range of 7.6–5.6 (Ludwig et al., 2003; Ishii et al., 2005), however, they also can be modulated by other endogenous and exogenous molecules such as lipids (Murakami et al., 2004; Wang et al., 2004) and synthetic ligands (Ludwig et al., 2003). Upon activation they engage different intracellular signaling pathways linked to the function of different G proteins, including stimulation of inositol phosphate or cAMP production. Due to their ability to sense extracellular acidification, different studies have investigated their role in inflammatory mouse models. The expression of GPR4, GPR65, and GPR132 is upregulated in several mouse models of inflammatory pain (Chen et al., 2009; Dai et al., 2017). Several studies have shown that ablating the function of these receptors, by pharmacological or transgenic approaches, reduces persistent pathological pain from inflammatory and neuropathic origin (Dai et al., 2017; Hsieh et al., 2017; Miltz et al., 2017). However, although their role as acid sensors and their involvement in inflammatory conditions have been demonstrated, their expression in sensory fibers innervating the ocular surface has never been determined, and therefore, their role in the detection of acidic insults and inflammatory conditions affecting the cornea and conjunctiva remains to be studied.

#### 3.5.2 Purinergic P2X Receptors

Purinergic P2X receptors are another group of ion channels gated by ATP or other purinergic derivatives, but protons act as allosteric modulators modulating the activation and function of these receptors (Coddou et al., 2011). The potency of activation of P2X_2_ by specific agonists is enhanced 5- to 10-fold by acidification and even small changes in extracellular pH (7.1–7.2), enhance the response of P2X_2/3_ heteromeric receptors. In contrast, acidification inhibits most of the homomeric P2X receptors. In particular, in some studies, P2X_3_ is slightly inhibited by acidification but in other P2X subtypes, acidic pH exerts a dual effect, shifting the concentration-response curve to the right but increasing the current amplitude and activation time constant. Almost all P2X receptors have been detected in the trigeminal ganglia of both human and rodent species (Manteniotis et al., 2013; Flegel et al., 2015). P2X_3_ receptor is mainly expressed in sensory ganglia and mRNA and protein is found in the cell bodies of both small and large trigeminal sensory neurons but has the highest level of expression among smaller neurons, specially, in non-peptidergic IB4^+^ neurons (Staikopoulos et al., 2007). P2X_4_, P2X_5_, and P2X_6_ also show significant levels of expression in the trigeminal ganglia and lower levels are found for P2X_1_, P2X_2_ (Flegel et al., 2015). Despite the studies in trigeminal ganglion neurons, and like PS-GPCRs, there is a lack of specific studies on purinergic receptors in the sensory nerve endings innervating the anterior part of the eye (cornea, sclera and conjunctiva) thus the role of protons modulating purinergic signaling remains to be properly studied.

### 3.6 Ion Channels in Ocular Non-Neuronal Cells

In addition to the proton-sensing ion channels expressed in peripheral nerves innervating the cornea or the conjunctiva, cells from the corneal epithelium, endothelium or the conjunctiva also express ion channels with important functions for ocular physiology. Members of the TRP family have also been identified in corneal cells, including TRPV1-4, TRPM8, TRPA1, or TRPC4 (Mergler et al., 2014). TRPV1 has been found in the epithelium, stroma and endothelium of the cornea (Yang et al., 2013a; Mergler et al., 2014). In the epithelium, TRPV1 activation leads to an increase in intracellular calcium that induces inflammatory cytokine release through MAPK (mitogen-activated protein kinase) signaling (Zhang et al., 2007), thus it appears that TRPV1 has a significant role in infiltration of inflammatory mediators in the corneal epithelium and stroma. The channel has been also involved in cell migration and proliferation, thus promoting corneal epithelial wound healing response. Because TRPV1 is sensitive to protons, it is likely that acidification contributes to ocular surface inflammation though this channel, promoting the release of interleukins and other inflammatory mediators.

## 4 Acidic Substances and Commercial Drugs

A chemical injury with an acidic substance on the ocular surface is a medical emergency that must be evaluated and treated immediately. The treatment is usually based on reestablishing corneal clarity, recovering the ocular surface and avoiding increased intraocular pressure and damage to the optic nerve to prevent visual impairment. In contact with the cornea, acidic substances (pH < 4) denature and precipitate proteins, and their coagulation produces the opacity of the cornea that characterizes severe acid burns. After the acidic injury, the recovery phase begins in which the corneal epithelium and the stroma are restored, inflammatory mechanisms become evident on the ocular surface and there is stromal ulceration and corneal scarring (Singh et al., 2013). The main early signs of an acid burn in the eye include ocular pain and irritation, increased tear secretion, swollen eyelids and blurred vision. TRPV1 and ASICs, activated by low pH, are the main channels that mediate eye pain after acid injury. Hence, decreased expression or blocking of TRPV1 reduces pain caused by chemical injuries at the cornea (Moreno-Montañés et al., 2018; Hatta et al., 2019). In addition to mediate pain responses, TRPV1 is involved in the release of proinflammatory cytokines after an injury of corneal epithelial cells. Therefore, it is a good candidate to control eye pain in corneal injuries and inflammatory responses in the wound healing process (Yang et al., 2013b). As mentioned above, TRPA1 has also been associated with corneal regeneration after chemical injuries (Okada et al., 2015).

A number of compounds used to treat eye diseases are formulated in acidic solutions to facilitate their solubilization and absorption through the cornea. Ocular topical application of these drugs can cause adverse side effects associated with irritation and toxicity of the corneal surface (Zhang et al., 2019). Dorzolamide hydrochloride (Trusopt^®^ as tradename), which is a carbonic anhydrase inhibitor indicated to treat ocular hypertension and primary open-angle glaucoma, has a pH value of 5.6 and usually causes ocular irritation in patients (Konowal et al., 1999; Gordon et al., 2008). Other compounds with low pH values used as commercial ophthalmic eye drops to treat glaucoma are the non-selective β-blocker levobunolol hydrochloride (Betagan^®^; pH 6.5) and the prostaglandin analog latanoprost (Xalatan^®^, Mylan^®^, Travatan^®^, Saflutan^®^, Cosopt^®^; pH values between 5.6 and 6.7). It is widely known that they cause temporary burning, redness, itching and blurred vision (Thygesen, 2018). Consistent with this, pH neutralization in some ophthalmic compounds abolishes ocular irritation associated with their use (Loftsson et al., 2012). In this sense, previous studies have shown that ASICs participate in the nociceptive responses produced by Mylan^®^ and Betagan^®^, and probably also by Trusopt^®^ (Callejo et al., 2015). The low pH of this last compound, as well as its lower osmolarity and high viscosity might involve the activation of TRPV1 and other mechanisms, thus ASIC blockers are not sufficient to decrease its irritative effect (Callejo et al., 2015). At this point, identification and characterization of ion channels and the molecular mechanisms mediating ocular discomfort caused by ophthalmic drugs is essential to try to avoid undesirable side effects.

## 5 Summary

The ocular surface is a particular structure of the body greatly exposed to external environment and to many irritative and painful stimuli. This is probably the reason why the cornea presents the highest sensory innervation of the body, which allows to detect potentially damaging stimuli and to respond accordingly with protective behaviors such as blinking or tearing. Several mechanical, chemical or thermal stimuli are known to activate corneal, conjunctival and scleral peripheral terminals of trigeminal sensory neurons (particularly, nociceptors). Among them, acidic stimuli are known induce firing of polymodal nociceptors through activation of specific ion channels in these neurons. Part of these responses are mediated by members of the ASIC family, as about 2/3 of corneal sensory neurons present ASIC-like currents. Specifically, homomeric ASIC1a, ASIC3 and heteromeric ASIC1/3 channels have been identified (Callejo et al., 2015). These channels are activated by moderate acidifications (pH 7.2–6.6) that can occur in the ocular surface during inflammation or allergic conditions, in addition to insults from external acidic solutions. In this sense, ASIC also contribute to nociceptor sensitization and pain during allergic keratoconjunctivitis, as blockade of ASIC3 channels diminish nocifensive behavior in rodent models (Callejo et al., 2015). Interestingly, in addition to protons, other compounds such as GMQ is able to activate ASIC3 in the ocular surface, inducing nocifensive behaviors (blinking and tearing), as well as firing of ocular sensory nerve fibers. Other members of the family such as ASIC1b, ASIC2a and ASIC2b are also probably present in ocular nociceptors, as expression has been found in the trigeminal ganglion (Callejo et al., 2015). Nevertheless, no clear identification in ocular sensory neurons has been provided to date.

Another channel long involved in acid sensing is TRPV1, as this channel is directly activated by protons (Tominaga et al., 1998). Despite moderate acidifications (pH 6–7) enhance the responses to capsaicin and heat, the channel needs stronger acidifications (pH < 6) to be directly gated by protons (Tominaga et al., 1998). These biophysical properties seem to indicate that it is unlikely that acidification occurring during ocular inflammation or allergy can directly activate the channel but, certainly, can enhance its response to heat or other compounds, thus intensifying painful sensations. In fact, blocking TRPV1 or TRPA1 with specific antagonists or siRNAs has been demonstrated to reduce polymodal nociceptors activity and ocular surface irritation by exogenous compounds or during allergic keratoconjunctivitis (Luna et al., 2007; Acosta et al., 2013). Besides, ASICs seem more prone to mediate the acidic responses to moderate acidifications. Nevertheless, important exogenous acidic insults are likely to activate both types of channels, thus a major activation of sensory nerve terminals will be achieved.

The contribution of other channels and receptors that are likely expressed in corneal or conjunctival nerve fibers are poorly studied to date. Some of these, such as K_2P_ channels can have a significant influence in the excitability of ocular sensory fibers, modulating their excitability and, in consequence, pain sensitivity. Because these potassium channels are polymodal integrators, like TRPV1 and TRPA1, different stimuli including protons, can modulate their activity to enhance or diminish nociceptive input.

Despite some of the mechanisms of proton sensing in the ocular surface are becoming to be elucidated, specific studies on the different types of channels or receptors involved are needed, as well as the different types of sensory fibers involved.
